# Halo-pelvic traction for extreme lumbar kyphosis: 3 rare cases with a completely folded lumbar spine

**DOI:** 10.1080/17453674.2020.1824170

**Published:** 2020-09-25

**Authors:** Yu Wang, Chunde Li, Long Liu, Longtao Qi

**Affiliations:** Department of Orthopaedics, Peking University First Hospital, Xicheng District , Beijing 100034, China

We present 3 patients who had extreme lumbar kyphosis and were treated with halo-pelvic traction (HPT) and anteroposterior fixation. In addition, we also describe a modified halo-pelvic apparatus that helps to relieve the discomfort and inconvenience of HPT.

Preoperative and postoperative data are summarized in Table 1, and radiographic measurements are given in Table 2.

**Table 1.** Intraoperative and postoperative results

Case no.	Age/sex	Levels fused	Days of traction	Estimated blood loss (L)	Operative time (h)	Height increase (cm)	Complications
1	30/F	T10–S2	70	1.6	8.1	8	Dural tear
2	40/F	T8–S1	42	1.1	6.6	6	None
3	23/F	T10–S2	46	0.4	7.4	6	None

**Table 2.** Radiographic follow-up

Case no.	1	2	3	Mean
Global kyphosis (°)				
Before traction	180	133	112	143
After traction	137	82	57	92
After lumbar surgery	88	18	56	54
2 years after surgery	84	20	55	50
2-year correction (%)	54	85	53	64
C7-sagittal vertical axis (mm)				
Before traction	74	148	65	96
After traction	–35	48	–18	–2
After lumbar surgery	36	39	–15	20
2 years after surgery	36	41	–12	21
Pelvic tilt (°)				
Before traction	48	47	35	43
After traction	11	21	26	20
After lumbar surgery	21	10	33	21
2 years after surgery	20	15	24	29
Sacral slope (°)				
Before traction	–37	–23	–23	–27
After traction	–14	–2	–18	–11
After lumbar surgery	–25	15	–20	–10
2 years after surgery	–22	16	–10	–5
Pelvic incidence (°)				
Before traction	7	23	13	14
After traction	8	20	9	12
After lumbar surgery	7	25	13	15
2 years after surgery	7	23	12	14

## Case 1

**Figure 1.**
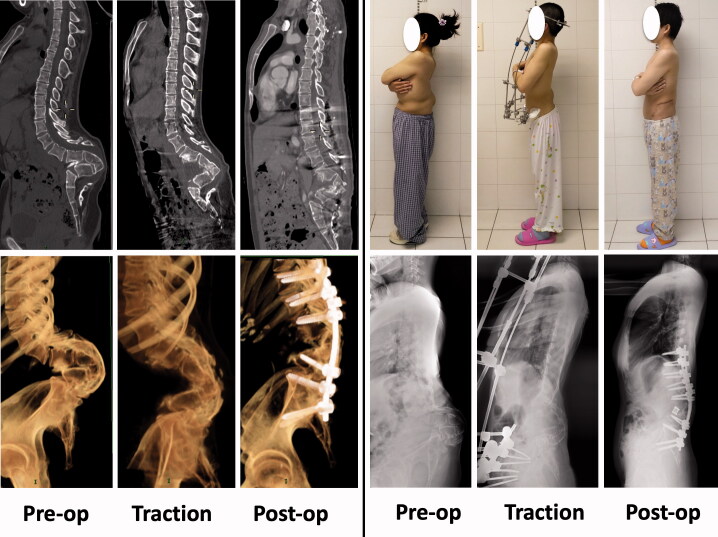
Case 1: Computed tomography (CT) revealed a congenital malformation in the lumbar region, and the lumbar spine appeared as if it was “completely folded up.” Treatment included HPT and anteroposterior fixation.

A 30-year-old female presented with a 10-year history of leg numbness and urinary incontinence (Figure 1). Physical examination showed a short trunk, and a bump in her lower back. CT revealed a congenital malformation in the lumbar region, and the lumbar spine appeared as if it was completely folded up. Considering the severity and complexity of the deformity, we decided to treat her in stages.

The first-stage operation consisted of posterior release and fixation of the halo-pelvic apparatus. Posterior release included spinous process resection and laminectomy. For the first week of HPT, the halo-pelvic frame was distracted at a rate of 1 cm per day. Beginning in the second week, the distraction rate was decreased to 0.5 cm per day. Notably, the patient described a sudden feeling of a “crack” on day 7 of traction. A lateral radiograph revealed evidence that her lumbar spine was “unfolding.” The second-stage operation was performed after 10 weeks of HPT. The procedure consisted of a vertebral osteotomy and pedicle screw fixation via a posterior approach, and mesh insertion via an anterior approach. She was 8 cm taller after surgery than before traction. By 6 months after the operation her leg numbness and urinary incontinence were relieved completely. No neurological or implant-related complications were noted over a 2-year follow-up period.

## Case 2

**Figure 2.**
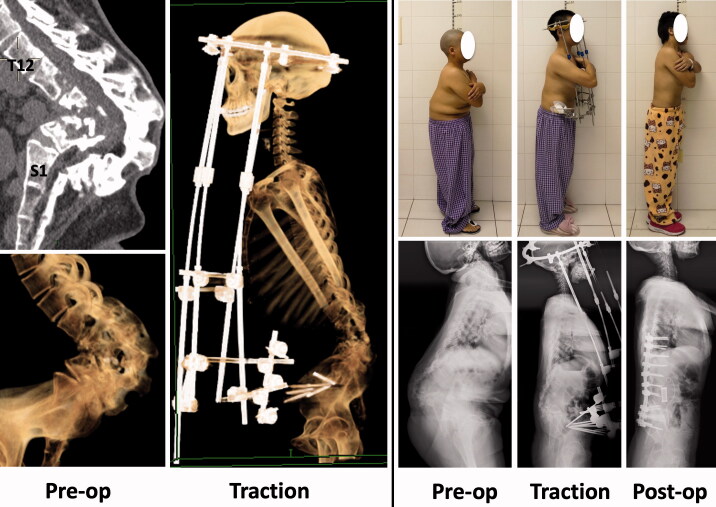
Case 2: CT revealed angular kyphosis in the lumbar region, almost complete absence of the vertebral bodies of L2 to L4, and complete fusion of the laminas of L1 to L5. Management consisted of HPT and anterior instrumentation and posterior fusion.

**Figure 3.**
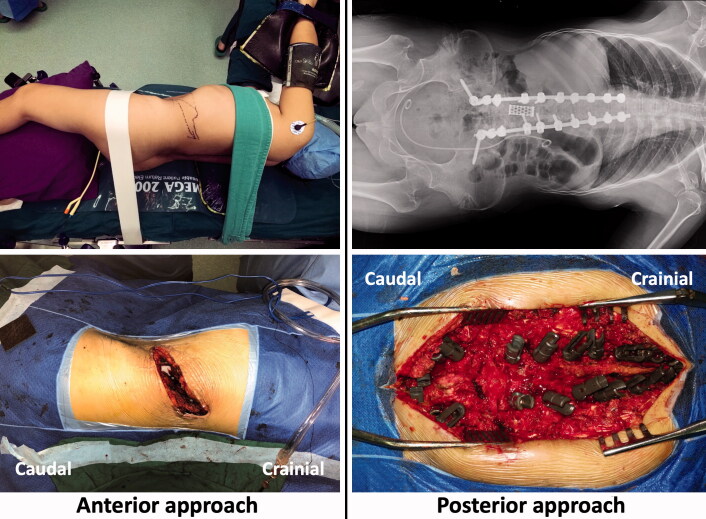
Case 2: Osteotomy and pedicle screw fixation were performed via a posterior approach and a mesh was inserted via an anterior approach during a single procedure.

A 40-year-old female presented with numbness and weakness of both lower extremities. She also complained of chronic low back pain, which she stated significantly impaired her quality of life. She had been diagnosed with spinal tuberculosis when she was 9 years old. CT revealed angular kyphosis in the lumbar region, almost complete absence of the vertebral bodies of L2 to L4, and complete fusion of the laminas of L1 to L5 (Figure 2). Her management consisted of 2 stages. The first-stage operation consisted of posterior release and fixation of the halo-pelvic apparatus. HPT was applied for 6 weeks, and the second-stage operation was then performed. This procedure consisted of anterior instrumentation and posterior fusion (Figure 3). She was 6 cm taller after surgery than before traction. During the 1st year after the second procedure the weakness and numbness of her lower extremities gradually diminished, and her low back pain was relieved. No neurological or implant-related complications were noted over a 2-year follow-up period.

## Case 3

**Figure 4.**
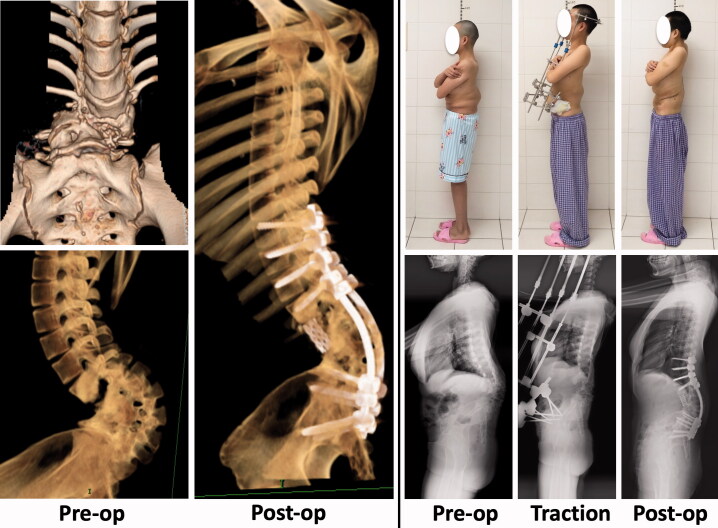
Case 3: CT revealed angular lumbar kyphosis, vertebral subluxation, absent vertebral bodies from L2 to L4 (presumably due to tuberculosis), and fusion of the L1–L5 laminas.

A 23-year-old female presented with chronic low back pain. She had been diagnosed with spinal tuberculosis when she was 2 years old. Physical examination showed a short trunk, and a bump in her lower back. CT revealed angular lumbar kyphosis, vertebral subluxation, absent vertebral bodies from L2 to L4 (presumably due to tuberculosis), and fusion of the L1–L5 laminas (Figure 4). Stage treatment was performed, similar to that used in Case 1 and Case 2. She began mobilization on day 5 after surgery, and was discharged to home on day 14 after surgery. She was 6 cm taller after surgery than before traction. She returned to work 6 months after surgery, and at that time her low back pain was relieved.

## Discussion

Spinal kyphosis is commonly due to ankylosing spondylitis, fractures, spinal tuberculosis, Scheuermann’s disease, degenerative spine disorders, and congenital deformities. Kyphotic deformities that require surgical treatment primarily occur in the thoracic, thoracolumbar, or lumbosacral region. In our experience, severe lumbar kyphosis is rare.

HPT was developed in the late 1960s by O’Brien et al. (1971). The powerful distraction forces generated by HPT can effectively correct various spinal deformities. The method was used extensively in the 1970s. However, its use began to decline after the 1980s because of its many drawbacks, including a long period of hospitalization, a multi-stage process, pain, discomfort, unflattering appearance, and various complications (Ransford and Manning 1975). In addition, advances in surgical techniques and methods of internal fixation rendered HPT obsolete.

Currently, most spinal deformities are treated by osteotomy and internal fixation (Suk et al. 2005, Lenke et al. 2010). However, treatment of extreme spinal deformities is challenging because a complex osteotomy and large-scale correction is required, and this significantly increases the incidence of neurological, vascular, and pulmonary complications. In addition, internal spinal stabilization can be difficult under some situations, such as severe rigid scoliosis, poor bone quality, revision surgery, respiratory dysfunction, and previously existing neurological deficits (Miladi 2013). HPT has been shown to increase the safety and correction rate for the surgical management of severe spinal deformities (Sponseller et al. 2008, Kim et al. 2009).

Herein, we report the treatment of 3 patients from 2017 with severe lumbar kyphosis. The patients were treated with HPT and anteroposterior fusion with good results, and follow-up periods of 2 years.

### Why was HPT necessary for the 3 patients?

Currently, spinal kyphosis is primarily treated with osteotomy and posterior fusion (Wang et al. 2015, Zhou et al. 2018, Hua et al. 2019, Zhang et al. 2019). However, performing a vertebral osteotomy via a posterior approach is difficult when a kyphosis is “folded” or the lumbar spine is collapsed. The procedure is difficult because all the nerve roots are compressed in a very small region of the posterior lumbar spine. The nerve roots block access for performing the vertebral osteotomy and mesh insertion; indeed, it is difficult to perform a vertebral osteotomy and mesh insertion through the limited space between any 2 lumbar nerve roots. Furthermore, the lumbar nerve roots are not as dispensable as some thoracic spine nerve roots. This problem can be solved by HPT, application of which spreads the nerve roots apart making it easier to perform a posterior osteotomy (Yu et al. 2016).

Another reason why HPT was necessary in these 3 patients is because there were few anchor points in the distal region of the implant construct. The lack of anchor points limits the corrective force of instrumentation. In all 3 patients, only 3 segments (L5 to S2) were available for screw insertion on the caudal side. As such, correction of the kyphosis with HPT helped to achieve better overall correction and prevent screw pull-out.

The deformities in the 3 patients presented were severe and complex, and direct correction would have been associated with significant risks. On the other hand, the gradual application of HPT for 6–10 weeks allowed reduction of the kyphosis, which decreased the risk of surgical complications. Furthermore, lengthening of the HPT frame was done with the patients conscious; therefore, any neurological deficits occurring as a result of the HPT would have been identified immediately. Lastly, the modified halo-pelvic device allowed the patients to move and lie more comfortably than if a traditional device had been used.

### How effective is presurgical HPT?

In our 3 patients, the average global kyphosis decreased from 143° before HPT to 92° after HPT. Obviously, a kyphosis of around 90° is much safer for surgery than one that is > 140°. Furthermore, the average global kyphosis further decreased to 54° after the final surgery.

In recent years, more attention has been given to sagittal spinal alignment during the correction of spinal deformities. Schwab et al. (2012) proposed that sagittal vertical axis (SVA), pelvic tilt (PT), and pelvic incidence–lumbar lordosis (PI–LL) mismatch are the 3 most important spinopelvic parameters, which should be carefully considered in the preoperative planning for the treatment of adult spinal deformities. To achieve good clinical outcome, realignment objectives have been reported to be an SVA < 50 mm, PT < 25°, and PI–LL equal to ±9°. More recently, Huang et al. (2020) reported that PT was the major radiographic contributor to Oswestry Disability Index (ODI) score in patients treated for ankylosing spondylitis (AS). The optimal sagittal alignment of AS patients who had undergone a single-level pedicle subtraction osteotomy (PSO) with a minimum 2-year follow-up was a PT < 24°. The PTs of our 3 patients presented here at 2-year follow-up were 20°, 15°, and 24°, respectively, which can be considered satisfactory outcomes according to the aforementioned criteria.

### A modified halo-pelvic apparatus

We used a modified halo-pelvic apparatus (WEGO) that helps to relieve the discomfort and inconvenience of HPT. Unlike the whole-ring pelvic frame in the traditional HPT, the modified pelvic frame is half-ring shaped, and thus there are no pins around the posterior pelvis. The modification increases patient comfort while they are lying on their back.

The pelvic ring and skull halo device are applied with the patient under general anesthesia. With the patient in the supine position, 3 pins (4.5 mm diameter) are inserted between the inner and outer table of the ilium on each side. The skull halo device is then placed. The HPT frame is not constructed at the time the pelvic and skull devices are placed. The frame is constructed 3–5 days later so that the patient has been able to accustom him/herself to the frame.

After the construction of the frame, distraction can be performed. For the first week, the frame is distracted at a rate of 1 cm per day. Beginning in the second week, the distraction rate is decreased to 0.5 cm per day. With these rates, the height of the patient can be increased by 8–10 cm in the first 2 weeks. Beginning in the third week, the distraction rate is decreased to 0.3–0.5 cm every 2–3 days depending on the patient’s condition and tolerance of the procedure. Traction is performed for 6–10 weeks in total, and the final surgery is then performed.

### Halo-pelvic traction versus halo-gravity traction (HGT)

Similar to HPT, HGT is another form of traction used to obtain correction of spinal deformity prior to operative treatment. It can also be applied to early-onset scoliosis as a delaying tactic. Compared with HPT, HGT is less cumbersome, because the pelvis and the legs remain unrestrained, encouraging patient mobility. Predictably HGT produces 30–35% correction of both coronal and sagittal plane deformity, which seems less effective compared with HPT. Meanwhile, HGT shows a lower complication rate (1–1.5% incidence of neurologic complication), which is safer than HPT.

In conclusion, HPT is useful in the management of extreme lumbar kyphosis. 6 weeks of HPT can greatly reduce the global kyphosis, and thus improve the conditions for corrective surgery.
